# Differential gut microbiota modulation associated with anti-Parkinsonian effects of *Ophiopogon japonicus* across geographical origins and extraction methods

**DOI:** 10.3389/fnut.2026.1907570

**Published:** 2026-08-06

**Authors:** Si-Jia Gao, Zi-Xuan Wang, Ai-Hua Xie, Jia-Xin Wu, Li-Yun Qiu, Tongkai Chen, Xiao-Jia Chen

**Affiliations:** 1 State Key Laboratory of Mechanism and Quality of Chinese Medicine, University of Macau, Macao SAR, China; 2 Institute of Chinese Medical Sciences, University of Macau, Macao SAR, China; 3 Chongqing Academy of Chinese Materia Medica, Chongqing University of Chinese Medicine, Chongqing, China; 4 State Key Laboratory of Traditional Chinese Medicine Syndrome, Science and Technology Innovation Center, Guangzhou University of Chinese Medicine, Guangzhou, China; 5 Macau Centre for Testing of Chinese Medicine, Institute of Chinese Medical Sciences, University of Macau, Macao SAR, China; 6 Department of Pharmaceutical Sciences, Faculty of Health Sciences, University of Macau, Macao SAR, China; 7 Zhuhai UM Science & Technology Research Institute, Zhuhai, China

**Keywords:** extraction method, geographical origin, gut microbiota, *Ophiopogon japonicus*, Parkinson's disease

## Abstract

**Introduction:**

*Ophiopogon japonicus* (OJ) is a medicine-food homologous plant widely consumed in East Asia and has attracted attention for its potential anti-Parkinsonian properties. However, the influence of geographical origin and extraction methods on its bioactive profile, gut microbiota modulation, and anti-Parkinsonian effects remain insufficiently understood.

**Methods:**

OJ samples were collected from two major producing regions (Sichuan and Zhejiang) and prepared using two extraction methods (water and ethanol). Chemical profiles were characterized by UHPLC-Q-TOF-MS. Anti-Parkinsonian effects were evaluated in 1-methyl-4-phenyl-1,2,3,6-tetrahydropyridine (MPTP)-induced PD mice through behavioral tests (pole, rotarod, open-field, gait, and Morris water maze), biochemical analyses (tyrosine hydroxylase, α-synuclein, and inflammatory markers), and histological examinations. Gut microbiota composition was analyzed via 16S rRNA gene sequencing to identify modulation patterns associated with health outcomes.

**Results:**

Chemical profiling revealed substantial compositional differences among extracts depending on geographical origin and extraction method. All OJ extracts significantly ameliorated MPTP-induced motor deficits, anxiety-like behaviors, dopaminergic neuron loss, and neuroinflammation, indicating broadly comparable anti-Parkinsonian effects. Notably, water extract of OJ from Sichuan (COJ_W) was the only group that significantly improved cognitive performance compared with the MPTP group. Concurrently, differential gut microbiota modulation patterns were observed across treatment groups. All OJ extracts significantly altered the abundance of *unclassified_c__Bacilli*, while COJ_W was characterized by a relative enrichment of taxa such as *Akkermansia, Dubosiella, Ileibacterium*, and *Christensenellaceae_R-7__group*.

**Conclusion:**

Geographical origin and extraction method influence the gut microbiota modulation patterns of OJ, which are associated with its anti-Parkinsonian effects across different functional domains. These findings highlight the importance of raw material selection and processing strategy in the functional evaluation of edible medicinal plants and provide insights into the complex relationship between gut microbiota modulation and health outcomes.

## 1 Introduction

Neurodegenerative diseases have garnered increasing attention as the global population becomes increasingly aged. Parkinson's disease (PD) is estimated to affect over 25.2 million individuals by 2050, according to an epidemiological study (1). Clinically, PD is characterized by the classic motor symptoms encapsulated by the acronym TRAP (tremor at rest, rigidity, akinesia/bradykinesia, and postural instability), often accompanied by prodromal non-motor manifestations such as constipation that may precede diagnosis by years. Beyond its profound impact on patients' quality of life, PD imposes a substantial socioeconomic burden. The classical pathogenesis of PD involves dopaminergic dysfunction caused by substantia nigra degeneration and abnormal folding of α-synuclein (α-syn), leading to progressive motor impairments (2). Levodopa (L-DOPA) remains the first-line pharmacotherapy for PD, while its long-time utility is frequently compromised by motor fluctuations, dyskinesia, and limited efficacy against non-motor symptoms. Therefore, complementary approaches based on dietary and functional food interventions are increasingly being explored as promising strategies for maintaining neurofunctional health.

Emerging evidence has redefined PD as more than a central nervous system disorder. According to Braak's hypothesis, misfolded α-syn aggregates may ascend to the brain from the enteric system through the vagus nerve. This mechanism is consistent with gut microbial signatures observed in PD patients. This microbial imbalance exacerbates intestinal permeability, leading to “leaky gut” and systemic inflammation, which ultimately accelerates dopaminergic neurodegeneration in the substantia nigra. Dysregulation of the gut microbiota and impaired intestinal barrier function are increasingly recognized as key contributors to PD pathogenesis, supporting PD as a gut-brain axis disease (3). Consequently, gut-targeted therapeutic strategies, including probiotics (4), prebiotics (5), and fecal microbiota transplantation (6), have been actively investigated and yielded promising outcomes (7), highlighting the potential of rebalancing intestinal microbial homeostasis in PD prevention and management.

Traditional Chinese medicine, distinguished by its holistic therapeutic profile, involving coordinated modulation of multiple targets and metabolic pathways, offers unique advantages in addressing complex neurodegenerative conditions. *Ophiopogon japonicus* (L. f.) Ker-Gawl. (OJ) is a representative herb with over two thousand years of clinical application in China and is officially recognized as a medicine-food homologous material. In addition to its medicinal use, OJ has long been consumed as a dietary ingredient in soups, teas, and health products, suggesting its safety for long-term oral intake and suitability for food-based intervention strategies. Traditionally, it is employed to nourish *Yin*, promote the production of body fluid, alleviate pulmonary dryness, and regulate cardiac heat to promote emotional calmness. Phytochemical analysis indicates that the major bioactive constituents of OJ include steroidal saponins, homoisoflavonoids, polysaccharides, and oligosaccharides. Pharmacological investigations have revealed that OJ exhibits the activity of anti-inflammatory, antioxidant, anticancer, immunomodulation, and neuroprotection (8–10). OJ extracts have also been reported to alleviate PD-related neurotoxicity through Notch pathway (11). Moreover, accumulating evidence indicates that OJ extracts can protect intestinal epithelial barriers and modulate gut microbiota under pathological conditions (12, 13), suggesting a functional role along the gut-brain axis.

However, the chemical composition and bioactivity of OJ may vary considerably depending on its geographical origin. The primary production areas of OJ in China are Sichuan and Zhejiang provinces, both of which produce geo-authentic medicinal material yet exhibit distinct phytochemical profiles. Previous comparative studies have shown that OJ from Zhejiang (ZOJ) is characterized by higher contents of homoisoflavonoids, while OJ from Sichuan (COJ) contains more specific saponin compounds (14, 15). In addition, different extraction solvents yield distinct bioactive constituents, thereby influencing functional properties and biological outcomes. Despite these recognized differences, few studies have systematically compared the biological functions of OJ from different origins or prepared with different extraction methods.

In the present study, ultra-high-performance liquid chromatography coupled with quadrupole time-of-flight mass spectrometry (UHPLC-Q-TOF-MS) was employed to characterize and compare the chemical profiles of OJ extracts. The anti-Parkinsonian and gut-modulatory effects of OJ from different geographical origins and extraction methods were evaluated using a 1-methyl-4-phenyl-1,2,3,6-tetrahydropyridine (MPTP)-induced murine PD model. Behavioral performance, cognitive function, neuroinflammation, and tyrosine hydroxylase-positive (TH^+^) neuron survival were assessed. Furthermore, 16S rRNA gene sequencing was employed to elucidate the potential mechanisms, with a particular focus on gut-brain axis regulation.

## 2 Materials and methods

### 2.1 Materials

Root tubers of OJ from Sichuan and Zhejiang were obtained from Guangdong Daxiang Chinese Medicine Pharmaceutical Co., Ltd. (Guangdong, China). The botanical identification was performed by the corresponding author. The voucher specimens were deposited at the Institute of Chinese Medical Sciences, University of Macau, Macau SAR, China. The MPTP was purchased from MedChemExpress (NJ, USA); anti-TH and anti-α-syn antibody were obtained from Abcam (Cambridge, UK); bicinchoninic acid protein assay kit was obtained from Beyotime (Shanghai, China). The following reference standards were purchased from Push BioTechnology (Chengdu, China): borneol 7-O-[β-D-apiofuranosyl-(1 → 6)]-β-D-glucopyranoside, ophiopogonin C, ophiopogonin Ra, ophiopogonside A, 14-hydroxy sprengerinin C, ophiopogonin D, ophiopogonin D', and cixiophiopogon A. Other reference standards were obtained from MUST BioTechnology (Chengdu, China): fructose, deacetyl ophiopojaponin A, methylophiopogonone A, methylophiopogonone B, methylophiopogonanone A, methylophiopogonanone B, 6-aldehydo-isoophiopogonone A, 6-aldehydo-isoophiopogonanone A, 8-formyl ophiopogonone B, ophiopogonanone C, 8-formyl ophiopogonanone B and ophiopojagonin C.

### 2.2 Preparation of OJ extracts

Samples of COJ and ZOJ were milled into powders prior to extraction. The extraction conditions were selected based on preliminary optimization experiments and traditional preparation practices. Each sample (200 g) was subjected to three cycles of reflux extraction for 2 h with absolute ethanol (ethanol content ≥ 99.7%, 1:10, w/v, 80 °C) or deionized water (1:10, w/v, 100 °C), respectively. After extraction, the combined supernatants were concentrated under reduced pressure and lyophilized to obtain COJ ethanol extract (COJ_E, yield: 1.79%, w/w), ZOJ ethanol extract (ZOJ_E, yield: 4.05%, w/w), COJ water extract (COJ_W, yield: 84.79%, w/w) and ZOJ water extract (ZOJ_W, yield: 83.24%, w/w).

### 2.3 UHPLC-Q-TOF-MS analysis of OJ extracts

Each lyophilized extract was reconstituted in its corresponding extraction solvent to a final concentration equivalent to 200 mg plant material/mL, then filtered using a 0.22 μm syringe filter before being injected into the LC-MS system. Chromatographic separation was conducted on a Waters ACQUITY™ ultra performance liquid chromatography (Waters Corp., Milford, MA, United States) by a Waters ACQUITY UPLC HSS T3 column (100 mm × 2.1 mm, 1.8 μm) at 35 °C with a flow rate of 0.4 mL/min. The mobile phase consisted of 0.1% aqueous formic acid (A) and acetonitrile (B). Gradient elution was performed as follows: 0–10 min, 10–25% B; 10–16 min, 25–30% B; 16–20 min, 30–40% B; 20–35 min, 40–45% B; 35–38 min, 45–55% B; 38–38.5 min, 55–60% B; 38.5–45 min, 60–70% B. The injection volume was 4 μL.

Mass spectrometric detection was conducted using a SYNAPT XS high-definition TOF mass spectrometer (Waters Corporation, Milford, MA, USA) operated in MS^E^ continuum mode. Analyses were conducted in both positive and negative electrospray ionization (ESI) modes. Instrument parameters were as follows: source temperature, 120 °C; desolvation temperature, 450 °C; cone gas flow, 50 L/h; desolvation gas flow, 900 L/h; capillary voltage, 3 kV for ESI(+) or −2.4 kV for ESI(–); sampling cone voltage, 40 V; and source offset voltage, 80 V. Mass acquisition range was 50–1,500 Da with a scan time of 0.2 s, and collision energy was set at 20–40 eV for high-energy acquisition. Mass accuracy was maintained via LockSpray™ using leucine enkephalin (200 pg/μL delivered at 5 μL/min) as reference compound, monitored at m/z 556.2771 for [M+H]^+^ in positive mode and m/z 554.2615 for [M^−^ H]^−^ in negative modes.

### 2.4 Determination of total carbohydrate content of OJ extracts

The total carbohydrate content of the OJ extracts was quantified by the phenol-sulfuric acid assay, employing fructose as the calibration standard. Briefly, 200 μL of the sample solution or fructose standard solutions were mixed with 100 μL of a 6% phenol solution, after which 0.5 mL of concentrated sulfuric acid was rapidly added. The mixture was shaken and incubated at 90 °C for 10 min to ensure complete color development. The absorbance was measured at 490 nm using an ultraviolet-visible spectrophotometer.

### 2.5 Animals

In this study, forty-two male C57BL/6J mice (8 weeks old) were acquired from the Guangdong Medical Laboratory Animal Center. Standard SPF housing conditions were provided, including a 12-h light/dark cycle, 25 ± 2 °C, and 55 ± 5% humidity, with food and water provided freely. Mice were allowed to acclimatize to the animal facility for 7 days before the start of the experiment. All experimental procedures were approved by the Animal Ethics Committee of Guangzhou University of Chinese Medicine (Approval No.: 20230813003) and performed in accordance with the ARRIVE guidelines.

### 2.6 Establishment of PD mouse model and treatment protocol

C57BL/6J male mice were randomly divided into 7 groups (*n* = 6): (1) control group, (2) MPTP group, (3) L-DOPA group (positive drug), (4)–(7) COJ_W, COJ_E, ZOJ_W, and ZOJ_E groups. To induce the PD model, MPTP (25 mg/kg/day) was administered via intraperitoneal injection for 5 successive days in all groups, with the exception of the control. The L-DOPA group received oral L-DOPA treatment at a dosage of 25 mg/kg. Extracts of OJ were prepared in saline as solutions or suspensions and administered at 1.82 g/kg of crude drug. The dose of OJ extract was selected according to the clinically recommended dose of OJ documented in the Chinese Pharmacopeia and converted to the mouse equivalent dose using the body surface area normalization method. Mice in the corresponding groups received daily oral gavage of COJ_W, COJ_E, ZOJ_W and ZOJ_E for 28 consecutive days. Mouse body weight was measured at 2-day intervals. At the end of the experiment mice were anesthetized with isoflurane, and blood samples were collected via the retro-orbital sinus. Tissues, including heart, liver, spleen, kidney, lung, and brain, were rapidly dissected on ice, and colonic contents were collected separately. All samples were then snap-frozen in liquid nitrogen and stored at −80 °C for further analysis.

### 2.7 Pole test

The experimental procedures were adapted from previous reports (16). Mice were positioned on a head-upward posture at a rough surface rod (1 cm diameter, 50 cm height), bradykinesia was assessed by measuring the time taken to turn downward (T-turn) and the total time taken to fully descend from the pole (T-total). Three trials were conducted for each animal.

### 2.8 Rotarod test

As described previously (17), mice were placed on a mouse rotarod apparatus (Ugo Basile, Italy) at a constant speed of 20 rpm for 2 min. During the 2-min test, drop frequency (drops) and the latency before falling (latency) were measured for each animal. Each mouse underwent the rotarod test three times, and analyses were conducted based on the average values obtained.

### 2.9 Open-field test (OFT)

To adapt to the surroundings, all animals rested in the testing room for at least 30 min before the trials commenced. Each mouse was placed alone in a square arena (60-cm^2^ arena with 40-cm walls) and allowed to explore freely for 10 min, and its locomotor trajectory was recorded by an overhead camera. During each session, the environment was kept dark and quiet, and arena was thoroughly cleaned with 75% ethanol between trials to eliminate excreta and odor traces.

### 2.10 Morris water maze (MWM)

The MWM test was conducted as described in our previous study (18). Mice received 5 days of training in a circular pool (160 cm diameter) with a hidden platform placed in the target quadrant. Food-grade titanium dioxide powder was pre-added to highlight the mice's silhouettes. For training days, the mice underwent three acquisition trials daily for finding the hidden platforms, and the swimming path and escape latency were recorded. If the platform was not found in 60 s, the mouse was guided to it and held there for 10 s. On the testing day, a 60-s probe trial was performed after removal of the hidden platform. Swimming trajectories, path length in the target quadrant, and the number of platform crossing were subsequently analyzed.

### 2.11 Gait analysis

Following the manufacturer's instructions, the DigiGait imaging system (Mouse Specifics Inc.) was adopted to analyze postural gait dynamics. Firstly, the mice were trained to walk on the transparent treadmill at a speed of 18 cm/s. Then, the gait of the mice walking continuously in the visual field was captured by the camera underneath. The portions of the paw, gait parameters, and relevant index values were identified by the software. The definition of the parameters of interest is depicted in Supplementary Table S1.

### 2.12 Hematoxylin and eosin (H&E) staining

After being fixed in 4% paraformaldehyde and embedded in paraffin, the tissue sections (5 μm thick) were stained with H&E following the standard protocol described previously (19). The liver, heart, spleen, lung, and kidney tissues were subjected to H&E staining to assess potential organ toxicity following OJ extract administration.

### 2.13 Immunofluorescence

Brain tissues were fixed, cryoprotected, embedded in optimal cutting temperature compound, and sectioned on a cryostat (Leica, Germany). After permeabilization and blocking, the sections were incubated with anti-TH primary antibody (1:1,000) at 4 °C for 12 h, followed by incubation with Alexa fluor 488-conjugated secondary antibody. Nuclei were counterstained with DAPI, and all images were acquired on a fluorescence microscope.

### 2.14 Western blot

Western blot analysis was performed according to a standardized protocol with minor modifications. Midbrain tissue lysates were prepared, separated by SDS-PAGE, and transferred onto PVDF membranes. After blocking, the membranes were incubated overnight at 4 °C with primary antibodies against TH (1:5,000), α-syn (1:1,000), and β-actin (1:3,000), followed by incubation with the corresponding HRP-conjugated secondary antibodies. Signals were developed using an enhanced chemiluminescence reagent (ChemiDoc, Bio-Rad).

### 2.15 Detection of proinflammatory factors

Commercial ELISA kits (Jianglai Bio, Shanghai, China) were used to measure interleukin (IL)-1β, IL-6, tumor necrosis factor (TNF)-α, and interferon (IFN)-γ levels in midbrain homogenates in accordance with the manufacturer's instructions.

### 2.16 Serum biochemical analysis

Biochemical parameters in serum were determined using a Beckman Coulter LH 780 Hematology Analyzer (CA, USA) according to the manufacturer's protocol.

### 2.17 Intestinal microbiota analysis

Because some animals had insufficient colonic content for reliable DNA extraction, only samples with adequate sample amount and sufficient DNA quality were included in the gut microbiota analysis. Finally, four samples per group were included for microbiota analysis. Microbial genomic DNA was extracted from the collected colonic contents, and purified amplicons were pooled in equal amounts and subjected to paired-end sequencing on an Illumina NextSeq 2000 platform (Illumina, San Diego, USA), conducted by Majorbio Bio-Pharm Technology Co., Ltd. (Shanghai, China) as previously described (20).

### 2.18 Statistical analysis

Microbial diversity and taxonomic composition were analyzed based on ASV abundance profiles. Alpha diversity indices, including Shannon, Chao, and Pielou_e, were calculated to assess microbial diversity, richness, and evenness. Beta diversity was assessed using Bray-Curtis distance-based PCoA. Taxonomic composition was compared among groups, and differentially enriched taxa were identified using LEfSe analysis. *P*-values were adjusted by the Benjamini-Hochberg method where appropriate.

Other data were presented as mean ± standard deviation (SD). Statistical analysis was performed using one-way ANOVA followed by Fisher's LSD *post hoc* test for pairwise comparisons among groups. A *P*-value < 0.05 was considered statistically significant. Statistical analyses were conducted using GraphPad Prism 10.0.

## 3 Results

### 3.1 Chemical analysis of OJ samples

**Figure 1:**
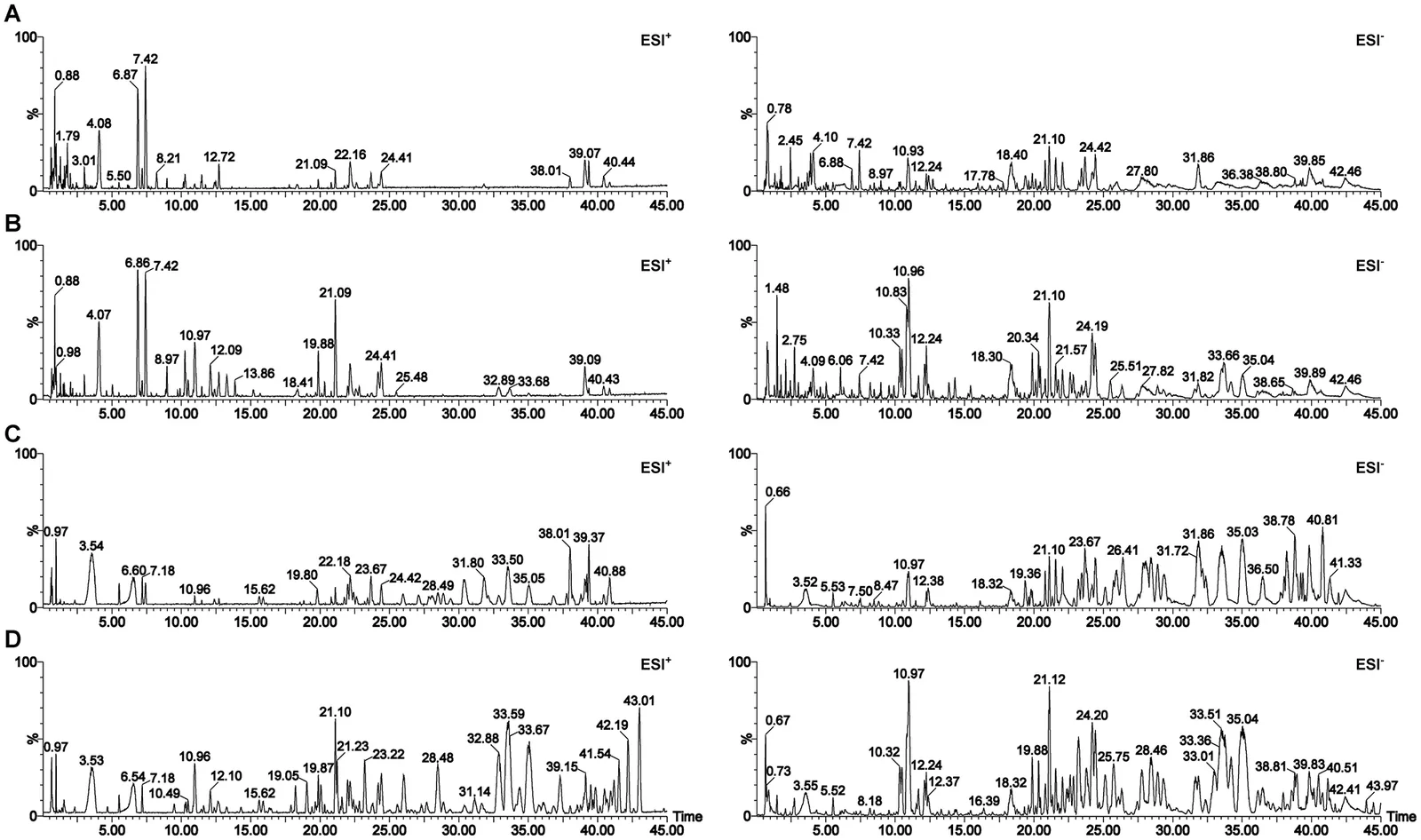
BPI chromatograms of **(A)** COJ_W, **(B)** ZOJ_W, **(C)** COJ_E, and **(D)** ZOJ_E obtained by UHPLC-Q-TOF-MS in ESI+ and ESI– modes. All BPI plots were displayed using the same intensity scale (max intensity = 6.00 e^6^).

Chromatographic profiles of samples from different geographical origins and extraction methods are shown in Figure 1. Chromatographic peaks were identified based on both reference standards and an in-house database, with detailed information provided in Supplementary Table S2. These compounds covered several major phytochemical categories, including saccharides, phenolic acids, homoisoflavonoids, and steroidal saponins, reflecting the chemical complexity of OJ.

Both extraction solvent and geographical origin affected the chemical profiles of OJ. Regarding extraction solvent, water extracts were characterized by early-eluting polar constituents (Rt < 8 min), such as saccharides (Peaks 2–3), phenolic and organic acids (e.g., Peaks 4–8 and 11), and lignan and phenolic glycosides (Peaks 9–10). In contrast, ethanol extracts showed enriched late-eluting secondary metabolites (Rt 19–45 min), dominated by steroidal saponins (e.g., Peaks 23–34, 36–37, 39–40, 42–43, 45–49, 55) and homoisoflavonoids (e.g., Peaks 35, 38, 41, 44, 50–54, 56). Total carbohydrate content analysis further supported these observations: COJ_W, COJ_E, ZOJ_W and ZOJ_E contained 87.1%, 48.6%, 87.7%, and 56.7% (w/w) carbohydrate, respectively, confirming that water extracts contained substantially higher levels of saccharides, whereas ethanol extracts exhibited greater chemical diversity in secondary metabolites.

Geographical origin also contributed to chemical variability under the same extraction solvent. Compared with ZOJ, COJ consistently exhibited fewer detectable chromatographic peaks in both water and ethanol extracts, suggesting lower chromatographic complexity. In water extracts, ZOJ presented more peaks in the mid-retention region (Rt 8–19 min), where steroidal saponins (e.g., Peaks 13–16 and 18–23) and monoterpenoid glycosides (e.g., Peak 17) are mainly eluted. Similarly, in ethanol extracts, ZOJ displayed a more crowded peak distribution in the late-eluting region (Rt 19–45 min) corresponding to steroidal saponins and homoisoflavonoids, whereas COJ showed a comparatively simplified profile.

Collectively, these results demonstrated that both geographical origin and extraction method contributed to the chemical diversity and compositional variation of OJ samples, which may contribute to differences in their functional properties.

### 3.2 OJ ameliorated the MPTP-induced PD movement disorders

**Figure 2:**
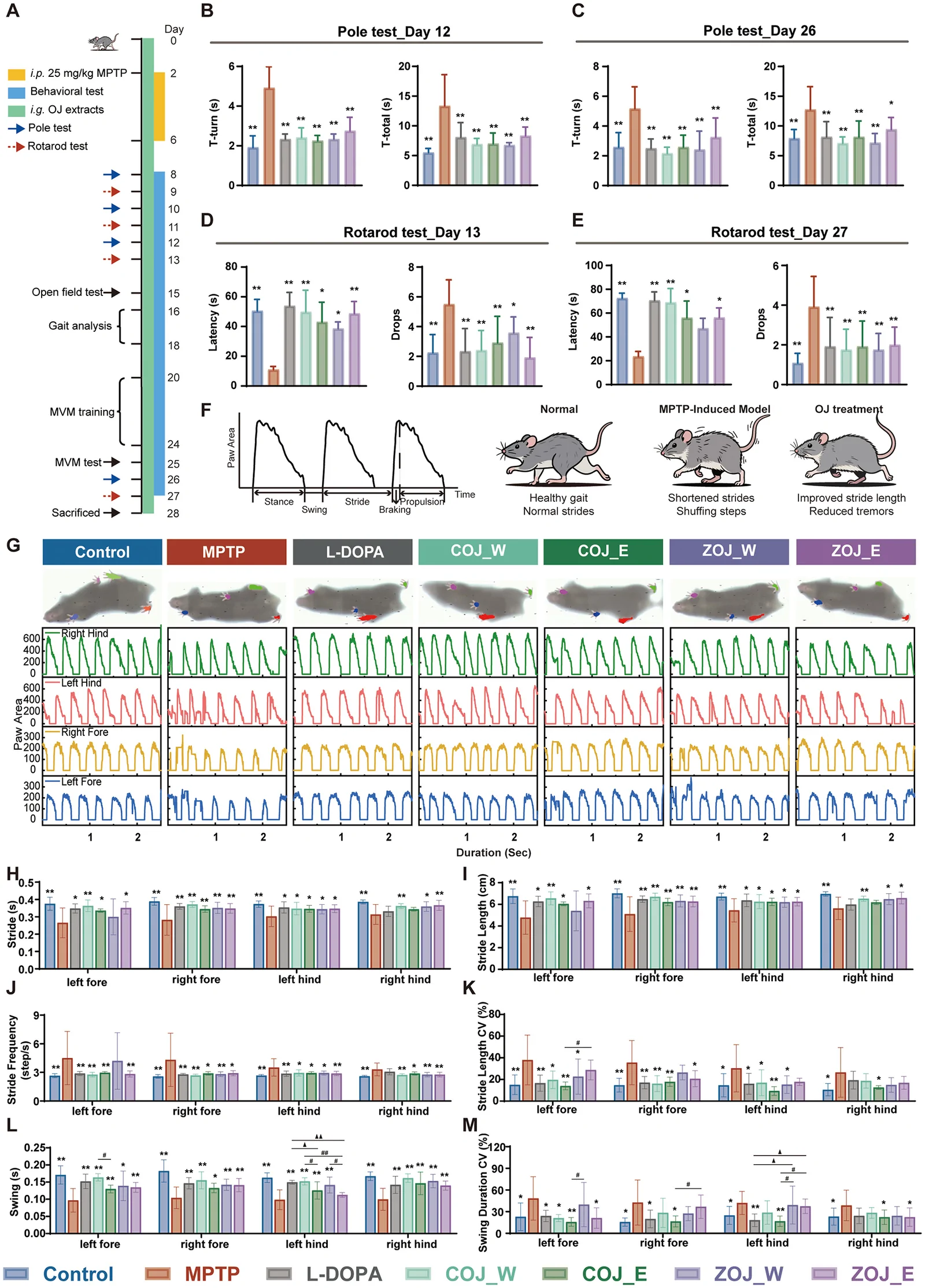
OJ extracts ameliorated movement disorders in MPTP-induced PD mice (*n* = 6). **(A)** Graphical illustration showing the behavioral tests in this study. Pole test on **(B)** day 12 and **(C)** day 26. Rotarod test on **(D)** day 13 and **(E)** day 27. **(F)** Schematic diagram of the gait analysis. **(G)** Typical paw placement patterns (top) and temporal gait rhythms (bottom) of the experimental mice captured via gait analysis. **(H)** Gait parameters including stride, **(I)** stride length, **(J)** stride frequency, **(K)** stride length CV, **(L)** swing, and **(M)** swing duration CV in mice. Compared to the MPTP group: *P < 0.05, **P < 0.01; compared to the L-DOPA group: ^▴^*P* < 0.05, ^▴▴^*P* < 0.01; comparison between OJ-treated groups: ^#^*P* < 0.05, ^##^*P* < 0.01.

To evaluate the anti-Parkinsonian effects of OJ extracts, we established a mouse model via intraperitoneal injection of MPTP. The overall experimental schedule is presented in Figure 2A, and motor performance was assessed as an initial outcome. No obvious toxicity was observed following OJ administration. Body weight monitoring, histopathological examination, and serum biochemical analyses revealed no treatment-related adverse effects (Supplementary Figures S1, S2), supporting the safety of OJ extracts at the tested doses. To eliminate unfamiliarity of mice with the behavioral test, mice were trained for the pole test and rotarod test from days 8 to 11. These tests were then carried out on days 12, 13, 26, and 27 to monitor whether motor deficits persisted throughout the study. As shown in Figures 2B, C, MPTP-induced mice showed the longest T-turn and T-total during the entire behavioral experiment, indicating significant motor disorders (*P* < 0.01). Treatment with L-DOPA significantly shortened T-turn and T-total compared with the MPTP group (*P* < 0.01). Similarly, both water and ethanol extracts of COJ and ZOJ showed improvements in bradykinesia and motor initiation deficit (*P* < 0.01, *P* < 0.05). Results from the rotarod test (Figures 2D, E) showed that MPTP administration caused a significant increase in the number of falls and a significantly shortened fall latency (*P* < 0.01). L-DOPA treatment significantly improved motor coordination, as indicated by prolonged fall latency and reduced numbers of falls (*P* < 0.01). And all treatment groups showed significant improvements in coordination function as well (*P* < 0.01, *P* < 0.05).

Considering that the pole and rotarod tests might be influenced by factors such as cognition and stress, we employed the gait test to provide a more refined assessment of motor function, specifically evaluating limb coordination and stride parameters (21). Consistent with previous reports, MPTP-induced mice exhibited obvious gait disturbances (Figures 2F, G), characterized by uncoordinated and asymmetric rhythm between steps, suggesting limb rigidity and postural control dysfunction, corresponding to the “gait freezing” in PD patients (22, 23). Statistical analysis of multiple gait parameters revealed abnormalities similar to those in PD patients. MPTP-treated mice showed stride abnormalities: decreased stride time and stride length (*P* < 0.01; Figures 2H, I), alongside increased stride frequency and stride length coefficient of variation (CV, *P* < 0.01, *P* < 0.05; Figures 2J, K). Swing abnormalities included shortened swing time and an increased swing duration CV (*P* < 0.01, *P* < 0.05; Figures 2L, M), reflecting loss of coordination and muscle rigidity, which are precursor indicators of freezing gait (24, 25). After intervention with L-DOPA or various OJ extracts, stride time and stride length were significantly increased compared to the model group, while stride frequency and stride length CV were significantly decreased (*P* < 0.01, *P* < 0.05; Figures 2H–K). Furthermore, swing time was prolonged after L-DOPA and OJ administration, and the swing duration CV was significantly reduced (*P* < 0.01, *P* < 0.05; Figures 2L, M). Further comparisons showed that the effects of most OJ extracts on stride frequency were comparable to those of L-DOPA. Significant differences between L-DOPA and certain OJ-treated groups were observed in swing-related parameters, particularly in the hindlimbs. Significant differences were also detected among OJ-treated groups in stride length CV, swing time, and swing duration CV, suggesting extract-specific effects on gait stability and limb coordination. Collectively, these results demonstrated that OJ extracts effectively attenuated MPTP-induced motor dysfunction, indicating anti-Parkinsonian potential in this model.

### 3.3 OJ attenuated the anxiety state and cognitive function in PD mice

**Figure 3:**
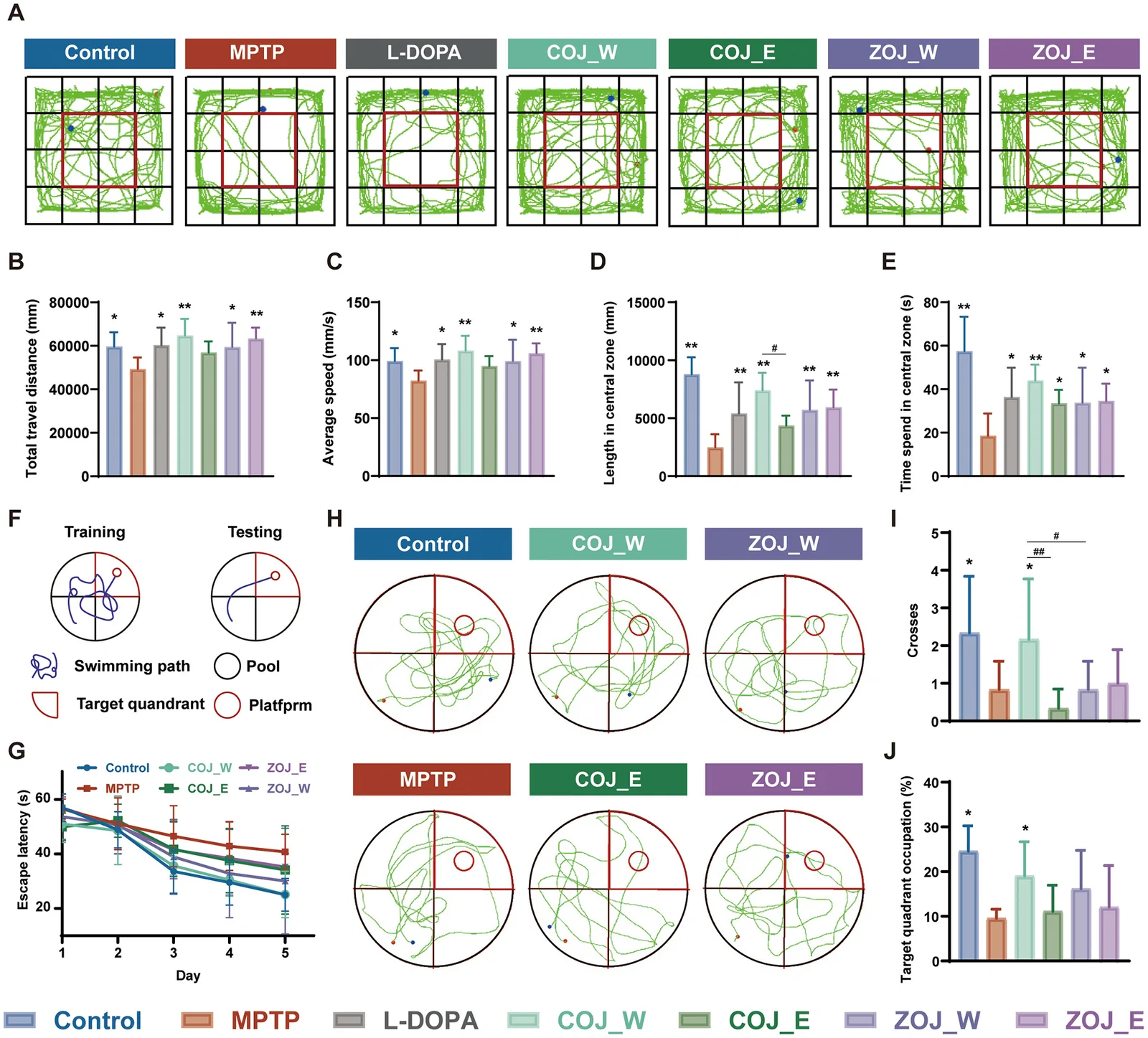
OJ attenuated the anxiety state and cognitive impairment in MPTP-induce PD mice (*n* = 6). **(A)** The representative movement trajectories of mice in the OFT. **(B)** Travel distance, **(C)** average speed, **(D)** length in central zone and **(E)** time spent in central zone of OFT in different groups of mice. **(F)** Schematic diagram of the MWM test. **(G)** Escape latencies during the acquisition phase of the MWM task for the various treatment groups. **(H)** Representative movement trajectories of mice in the MWM task. **(I)** The platform crosses time and **(J)** distance traveled in the target quadrant of mice in different treatment groups in MVM test. Compared to the MPTP group: *P < 0.05, **P < 0.01; comparison between OJ-treated groups: ^#^*P* < 0.05, ^##^*P* < 0.01.

Spontaneous movement and anxiety-like behavior in mice were evaluated using the OFT (26). Figure 3A shows the 10 min movement trajectories of the mice. After MPTP administration, the movement trajectory was significantly reduced, the total travel distance markedly decreased (*P* < 0.05; Figure 3B), and the average speed also significantly declined (*P* < 0.05; Figure 3C). Compared with the control group, both travel length and time spent in the center were significantly decreased (*P* < 0.01; Figures 3D, E). These findings indicated that the MPTP model not only reduced spontaneous locomotion but also induced anxiety-like behavior, decreasing exploratory activity. This is because mice naturally prefer staying near the chamber walls (27). L-DOPA treatment significantly restored spontaneous locomotor activity and exploratory behavior, as reflected by increased total travel distance, average speed, central zone travel length, and time spent in the central zone (*P* < 0.01, *P* < 0.05). Similarly, OJ extracts, except COJ_E in some parameters, effectively reversed MPTP-induced deficits in locomotor activity and anxiety-like behavior (*P* < 0.01, *P* < 0.05), demonstrating the recovery of spontaneous locomotion and amelioration of anxiety-like behavior.

Beyond motor symptoms, PD individuals frequently suffer from diverse cognitive challenges, ranging from weakened learning memory performance and visuospatial acuity to the more severe stages of dementia (28, 29). To quantify cognitive performance, mice underwent MWM testing, which involved a 5-day training focused on remembering the location of a hidden platform beneath the water surface and a final test to evaluate memory stability (Figure 3F). During the training phase, the escape latency gradually decreased across days for all groups; however, the latency decreased most slowly in MPTP-induced PD mice, indicating impaired learning ability compared to the control group (Figure 3G). Representative movement trajectories of mice from each group on the final test day are shown in Figure 3H. Control group mice frequently traversed the former platform site; in contrast, the MPTP group rarely reached it. The COJ_W group exhibited significantly more platform crossings compared to the MPTP mice (*P* < 0.05; Figures 3H, I). Additionally, the path length within the target quadrant (marked by a red frame) was measured. Similarly, Figure 3J shows a marked decline in the distance traveled in the target quadrant by the MPTP-treated mice (*P* < 0.05), and this reduction could be ameliorated by COJ_W. Further comparisons among OJ-treated groups showed that COJ_W significantly increased platform crossings compared with COJ_E and ZOJ_W (*P* < 0.01, *P* < 0.05). Interestingly, COJ_W was the only extract to significantly improve cognitive function, while other extracts had minimal effects, suggesting that different OJ extracts exert distinct functional effects on cognitive domains, shaped by geographical origin and extraction method.

### 3.4 OJ alleviated neuron viability and inflammation

**Figure 4:**
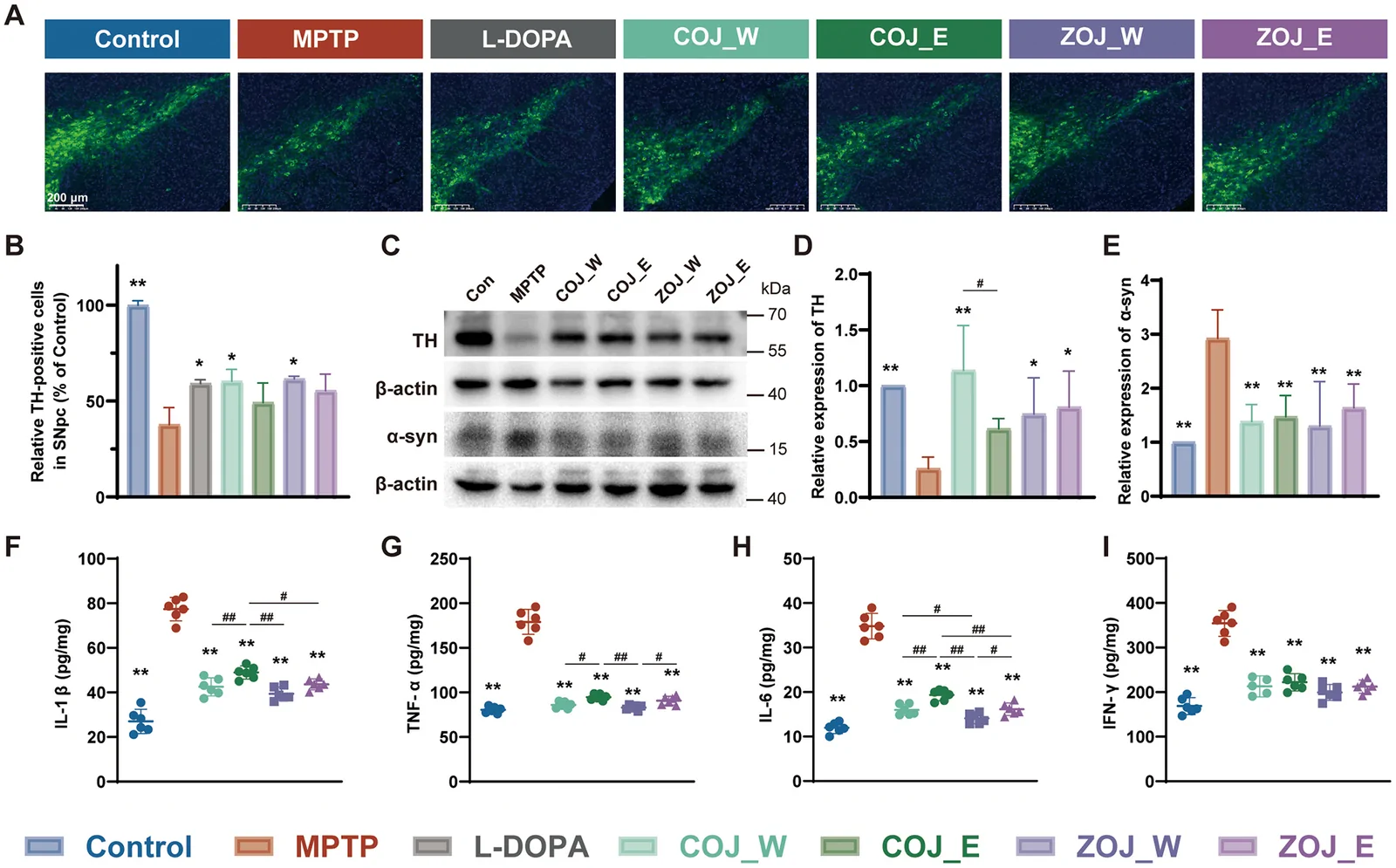
OJ alleviated neuron viability and inflammation. **(A)** Immunofluorescence visualization of TH^+^ dopaminergic neurons within the murine SNpc following various experimental interventions. Scale bar: 200 μm. **(B)** Quantitative analysis of TH^+^ neurons in SNpc (*n* = 3). **(C–E)** The protein expression of TH and α-syn in midbrain of mice in different treatment groups (*n* = 3). The levels of **(F)** IL-1β, **(G)** TNF-α, **(H)** IL-6, and **(I)** IFN-γ in the mice midbrain after different treatments (*n* = 6). Compared to the MPTP group: *P < 0.05, **P < 0.01; comparison between OJ-treated groups: ^#^*P* < 0.05, ^##^*P* < 0.01.

The core pathology of PD lies the toxic aggregation of misfolded α-syn. This protopathic process triggers a degenerative cascade within the substantia nigra pars compacta (SNpc), leading to progressive loss of dopaminergic neurons and subsequent depletion of striatal dopamine levels and motor deficits observed in the clinical. This study investigated the effects of OJ extracts on TH^+^ neurons in the SNpc on an MPTP-induced PD mouse model. Quantitative analysis of the midbrain sections (Figures 4A, B) revealed a marked depletion of TH^+^ neurons following MPTP challenge (*P* < 0.01). L-DOPA treatment significantly restored TH^+^ neuronal expression in the SNpc (*P* < 0.05).

Similar anti-Parkinsonian effects were observed in the COJ_W and ZOJ_W treatment groups (*P* < 0.05), whereas ethanol extracts did not produce statistically significant effects. Additionally, we quantified TH and α-syn protein expression in mice midbrain tissues. As expected, MPTP-induced mice exhibited significantly reduced TH (*P* < 0.01) and elevated α-syn (*P* < 0.01; Figures 4C–E). COJ_W, ZOJ_W and ZOJ_E effectively reversed the MPTP-induced reduction in TH expression (*P* < 0.01, *P* < 0.05), and all OJ extract-treated groups exhibited a remarkable decrease in α-syn level compared to the model group (*P* < 0.01). Studies have demonstrated abnormal aggregation of α-syn may lead to activation of immune cells within the central nervous system, leading to immune dysregulation and severe neuroinflammation (30). Thus, reducing α-syn levels may attenuate immune activation, neuroinflammation and rescuing dopaminergic neurons. We next measured pro-inflammatory cytokines in midbrain homogenates. The results (Figures 4F–I) revealed that MPTP treatment significantly elevated IL-1β, TNF-α, IL-6, and IFN-γ levels (*P* < 0.01), consistent with previous reports (30). Intervention with all OJ extracts resulted in a marked downregulation of these inflammatory cytokine levels toward the control group (*P* < 0.01), suggesting that OJ may ameliorate neuroinflammation and maintain cerebral homeostasis. Further comparisons among OJ-treated groups revealed extract-dependent differences in the regulation of IL-1β, TNF-α, and IL-6. In particular, COJ_E generally showed relatively higher cytokine levels than several other OJ-treated groups, whereas COJ_W and ZOJ_W exhibited stronger inhibitory effects on these inflammatory mediators (*P* < 0.05, *P* < 0.01). No obvious intergroup difference among OJ extracts was observed for IFN-γ.

### 3.5 OJ normalized the gut microbiota in PD mice

**Figure 5:**
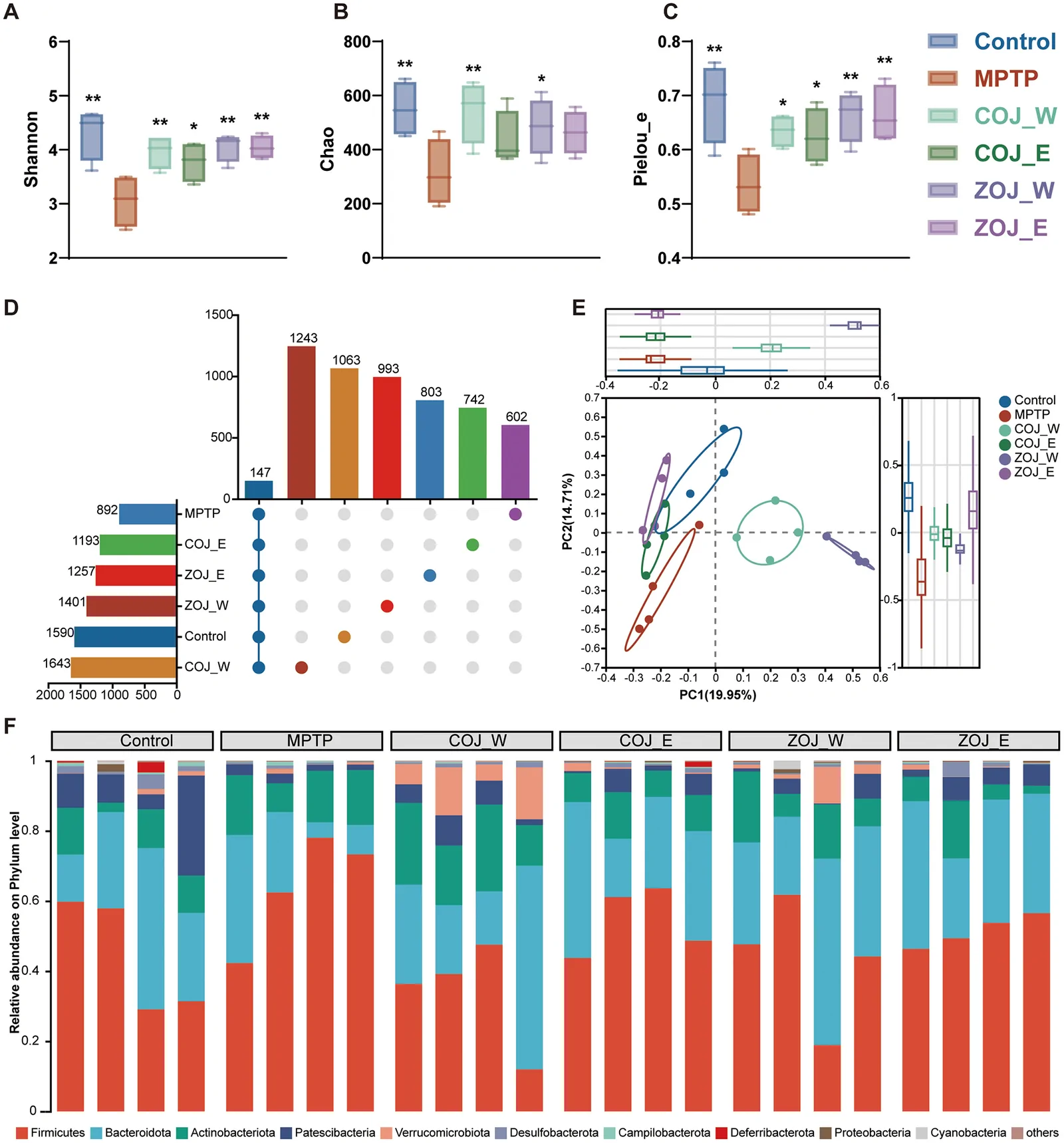
OJ normalized the gut microbial profile in PD mice (*n* = 4). **(A)** Shanno, **(B)** Chao and **(C)** Pielou_e index of mice gut microbiota at ASV levels. **(D)** UpSet plots at ASV levels. **(E)** PCoA of mice gut microbiota at ASV level. **(F)** Bar plots of the phylum abundance. Compared to the MPTP group: *P < 0.05, **P < 0.01.

Given the emerging role of the gut microbiota in PD-related neurodegeneration (31), targeting gut microbial perturbations has emerged as a viable intervention strategy. The discovery of the gut-brain axis represents a novel breakthrough for treating neurological disorders. Consequently, we performed 16S rRNA sequencing to monitor microbial variations of MPTP-induced PD mice following treatment with OJ extract. Alpha diversity analysis (Figures 5A–C) showed that MPTP significantly induced the reduction of community diversity (Shannon), richness (Chao) and evenness (Pielou_e) in mice gut microbiota (*P* < 0.01). Intervention with COJ_W and ZOJ_W significantly restored all three parameters (*P* < 0.01, *P* < 0.05), while COJ_E and ZOJ_E improved community diversity and evenness (*P* < 0.01, *P* < 0.05). These results suggest that OJ extracts partially ameliorated the reduction in gut microbial diversity induced by MPTP. UpSet plots showed that 147 amplicon sequence variants (ASVs) were shared across all groups (Figure 5D). To visualize the structural divergence and inter-group similarities, we utilized principal coordinates analysis (PCoA) to map the microbial community landscape across all samples. PCoA showed a tendency of samples within the same group to cluster together (Figure 5E). Notably, a clear separation was observed between the MPTP and the control group, indicating a significant difference in their microbiota. Additionally, all treatment groups formed clusters distinct from that of the MPTP group. We then examined the microbial profiles of each group at phylum level. As shown in Figure 5F, Firmicutes and Bacteroidota were the dominant phyla in all groups, representing 70%-80% of the total microbial abundance. In the MPTP group, Firmicutes and Actinobacteriota increased in relative abundance, while Bacteroidota and Patescibacteria decreased. OJ extracts exhibited both similar and different modulatory effects on the gut microbiota: all OJ extracts effectively modulated the dysregulated abundances of Firmicutes and Bacteroidetes and increased the level of Patescibacteria. Notably, the abundance of Verrucomicrobiota was significantly elevated in the gut microbiota of mice treated with COJ_W and ZOJ_W.

**Figure 6:**
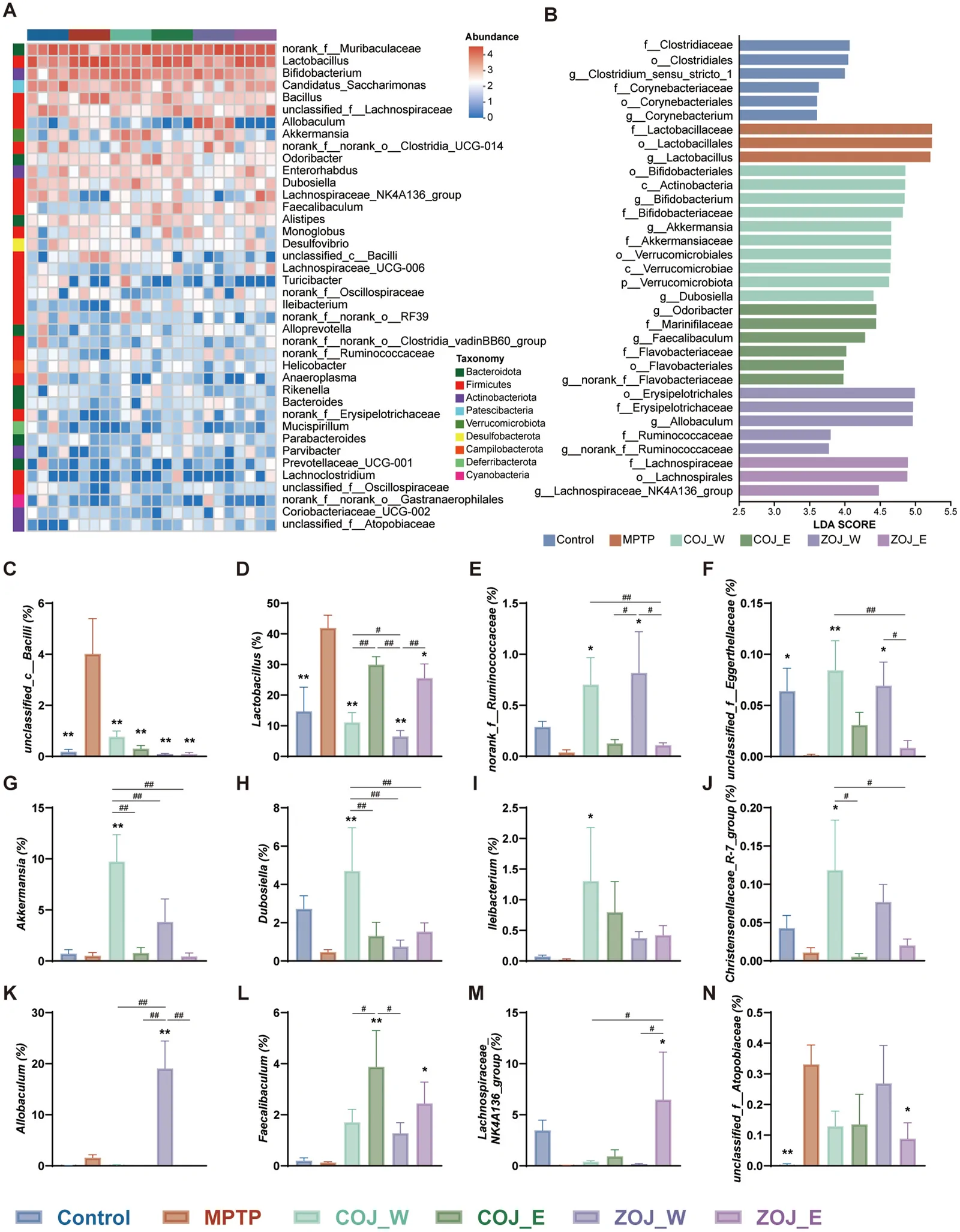
Different OJ extracts ameliorated gut dysbiosis and restored microbial homeostasis in PD mice (*n* = 4). **(A)** Heat map of relative abundance of 40 genera. **(B)** LEfSe analysis of mice microbiota with different treatment. Relative abundance of **(C)** *unclassified_c__Bacilli*, **(D)** *Lactobacillus*, **(E)** *norank_f__Ruminococcaceae*, **(F)** *unclassified_f__Eggerthellaceae*, **(G)** *Akkermansia*, **(H)** *Dubosiella*, **(I)** *Ileibacterium*, **(J)** *Christensenellaceae_R-7_group*, **(K)** *Allobaculum*, **(L)** *Faecalibaculum*, **(M)** *Lachnospiraceae_NK4A136_group* and **(N)** *unclassified_f__Atopobiaceae*. Compared to the MPTP group: *P < 0.05, **P < 0.01; comparison between OJ-treated groups: ^#^*P* < 0.05, ^##^*P* < 0.01.

Analysis at the genus level provided more detailed insight into microbial changes (Figure 6). Figure 6A shows a heatmap illustrating changes in gut microbiota at the genus level. The abundances of *Lactobacillus, Bacillus, Allobaculum*, and *unclassified_c__Bacilli* were increased, whereas *Candidatus_Saccharimonas, unclassified_f__Lachnospiraceae, Lachnospiraceae_NK4A136_group*, and *Dubosiella* were decreased in the MPTP group relative to the control group. COJ_W and ZOJ_W had more similar gut microbiota structures, while COJ_E and ZOJ_E also showed greater similarity, suggesting that OJ extracts prepared using the same extraction method resulted in similar gut microbiota structures in mice, rather than those from the same origin. LEfSe analysis was used to identify discriminative taxa across different levels (Figure 6B). Consistently, COJ_W group harbored a greater number of dominant bacterial taxa, with Verrucomicrobiota at the phylum level and *Akkermansia* at the genus level being predominant; these changes may be associated with its regulatory effects on PD. As shown in Figures 6C–N, the taxa with statistically significant differences among the six groups were summarized. *Unclassified_c__Bacilli* abundance was markedly elevated in PD mouse feces (*P* < 0.01), and all OJ extracts significantly reduced its levels (*P* < 0.01; Figure 6C). A similar trend was observed for *Lactobacillus*, with the water extracts showing stronger modulation (*P* < 0.01; Figure 6D). In addition, the OJ water extracts significantly increased *norank_f__Ruminococcaceae* and *unclassified_f__Eggerthellaceae* (*P* < 0.01, *P* < 0.05; Figures 6E, F). Dominant genera in the COJ_W group included *Akkermansia, Dubosiella, Ileibacterium*, and *Christensenellaceae_R-7_group* (*P* < 0.01, *P* < 0.05; Figures 6G–J). ZOJ_W significantly enhanced *Allobaculum* abundance (*P* < 0.01; Figure 6K). Both ethanol extracts increased *Faecalibaculum* abundance (*P* < 0.01, *P* < 0.05; Figure 6L), with ZOJ_E significantly elevated *Lachnospiraceae_NK4A136_group* and reduced *unclassified_f__Atopobiaceae* abundance in PD mice (*P* < 0.05; Figures 6M, N). Significant differences were also observed among OJ-treated groups for *Lactobacillus, norank_f_Ruminococcaceae, unclassified_f_Eggerthellaceae, Akkermansia, Dubosiella, Christensenellaceae_R-7_group, Allobaculum, Faecalibaculum*, and *Lachnospiraceae_NK4A136_group* (*P* < 0.01, *P* < 0.05). These findings suggest that OJ extracts derived from different geographical origins and extraction methods were associated with distinct alterations in gut microbiota composition. COJ_W was associated with a more pronounced increase in several taxa, such as *Akkermansia* and *Dubosiella*. However, the functional relevance of these microbial changes requires further validation.

### 3.6 Correlation analysis between gut microbiota, neuroinflammation, and behavioral performance

**Figure 7:**
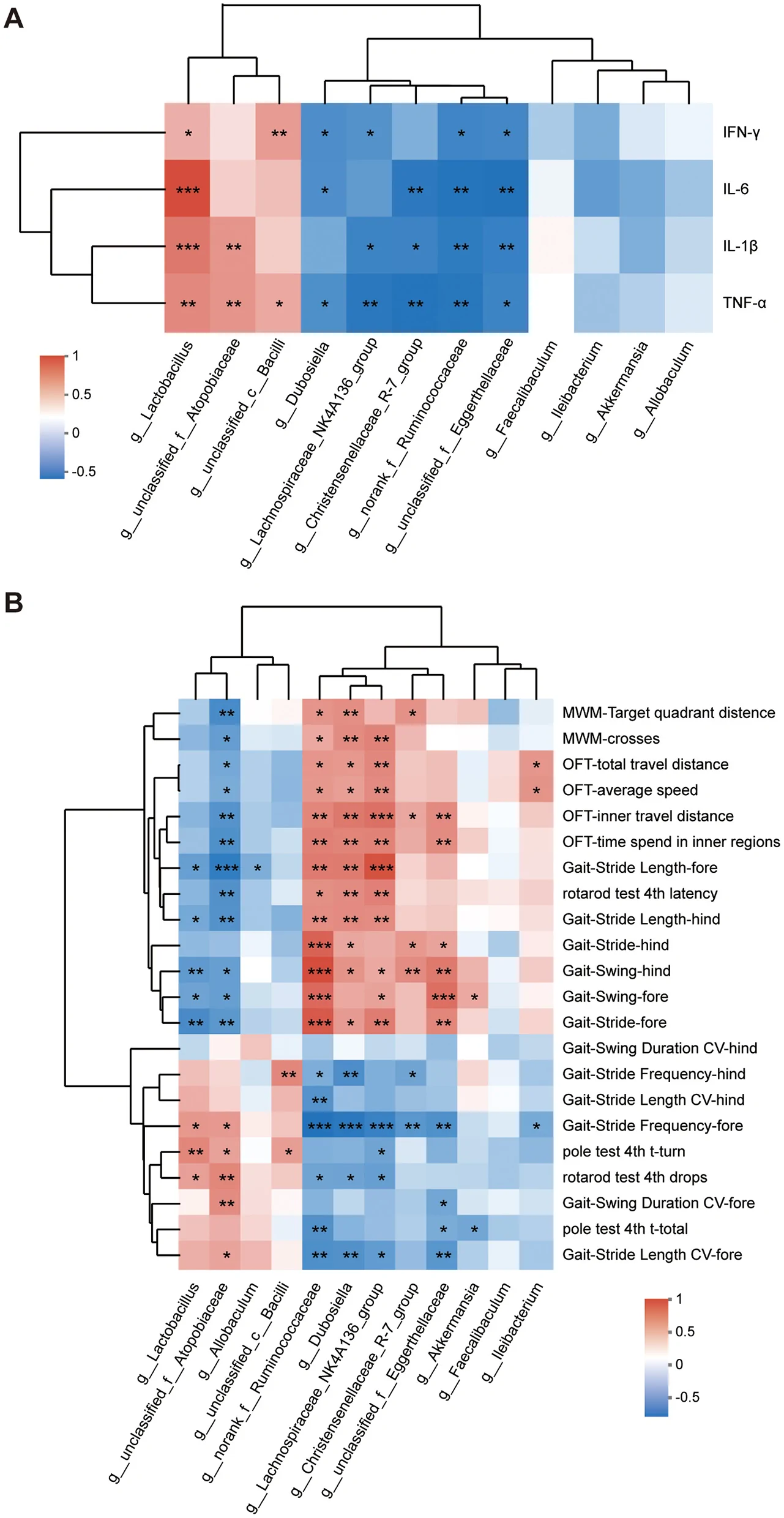
Spearman correlation analysis. Between differential bacterial genera and **(A)** neuroinflammatory cytokines and **(B)** behavioral parameters. *P < 0.05, **P < 0.01, ***P < 0.001.

To explore the associations between gut microbiota alterations and PD-related pathological changes, Spearman correlation analyses were performed between differential bacterial genera, neuroinflammatory cytokines, and behavioral parameters. As shown in Figure 7A, *Lactobacillus, unclassified_f_Atopobiaceae* and *unclassified_c_Bacilli* exhibited positive correlations with pro-inflammatory cytokines. In contrast, *Dubosiella, Lachnospiraceae_NK4A136_group, Christensenellaceae_R-7_group, norank_f_Ruminococcaceae*, and *unclassified_f_Eggerthellaceae* showed significant negative correlations with multiple inflammatory cytokines. Furthermore, correlation analysis with behavioral parameters revealed that *Lactobacillus, unclassified_f_Atopobiaceae*, and *unclassified_c_Bacilli* abundance was associated with behavioral impairments, whereas *norank_f_Ruminococcaceae, Dubosiella, Lachnospiraceae_NK4A136_group, Christensenellaceae_R-7_group*, and *unclassified_f_Eggerthellaceae* were generally associated with improved motor and exploratory performance (Figure 7B). These findings indicate potential associations among gut microbiota, neuroinflammation, and behavioral outcomes. Further studies are required to determine the mechanistic basis of these associations and to better understand the role of the microbiota-gut-brain axis in PD progression.

## 4 Discussion

The current investigation offers an in-depth comparison of the anti-Parkinsonian effects of OJ derived from different geographical origins and prepared using different extraction methods. Our findings showed that OJ significantly ameliorated motor impairments, alleviated anxiety-like behavior, enhanced cognitive performance, attenuated dopaminergic neuronal degeneration, and reduced neuroinflammation in an MPTP-induced PD mouse model. Among the tested extracts, COJ_W showed relatively greater improvement in cognitive performance. Mechanistic study suggested that these anti-Parkinsonian effects were closely associated with suppression of neuroinflammation and modulation of gut microbiota composition.

We employed a well-established subacute PD mouse model, which reliably induces typical motor disorders, neuronal loss in SNpc, and neurochemical alterations that closely mirror human PD pathology (32, 33). L-DOPA was used as a reference drug only in motor behavioral experiments. The behavioral assessments adopted in this study allowed a comprehensive evaluation of both motor and non-motor symptoms. The pole test primarily assesses core motor deficits in PD, namely bradykinesia and motor initiation deficit (34). However, the pole test alone cannot precisely distinguish bradykinesia from coordination and balance impairment, so the rotarod test was conducted subsequently. The rotarod test reflects the endurance of mice (26), while the pole test mainly focuses on the speed. Gait disturbances are one of the most prevalent clinical manifestations in PD patients. The hallmark characteristics include bradykinesia (slowness of movement), shuffling gait with reduced stride length, and postural instability. The characteristic reduction in stride length following MPTP administration closely approximates the shuffling gait in PD patients; this phenomenon presumably stems from acute dysfunction in the dopaminergic signaling of the nigrostriatal circuit. In this investigation, MPTP administration induced PD-like locomotor deficits, whereas intervention with OJ extracts significantly improved motor function. Clinical cohorts consistently highlight a high prevalence of profound non-motor comorbidities in PD, such as anxiety and cognitive decline. In this study, the OFT was employed to assess anxiety-like behavior, and the MWM was used to evaluate cognitive performance. Consistent with previous findings, the MPTP model resulted in increased anxiety levels and impaired cognitive function in mice. All OJ extracts except COJ_E demonstrated the ability to ameliorate anxiety-like behavior in mice. Notably, COJ_W exerted a relatively more pronounced improvement on cognitive performance, suggesting specific bioactive components in COJ_W may play a role in modulating neural processes related to learning and memory. In behavioral tests evaluating motor function, the efficacy of OJ treatment was comparable to that of the positive control, L-DOPA. However, although the MPTP-induced mouse model is widely used and reproduces key pathological and behavioral features of PD, it remains a toxin-induced model and cannot fully recapitulate the multifactorial etiology and progressive course of human PD. In addition, only male mice were included to minimize variability associated with estrous cycle-dependent hormonal fluctuations and to ensure the reproducibility of MPTP-induced lesions. However, sex-dependent differences in MPTP susceptibility, immune responses, and gut microbiota composition may influence the observed effects and should be considered in future studies.

The observed differences among OJ extracts may be linked to variations in their chemical composition. The LC-MS profiles indicated that both extraction solvent and geographical origin influenced the phytochemical composition of OJ extracts. Water extracts were enriched in polar constituents, whereas ethanol extracts contained higher levels of late-eluting secondary metabolites. Consistent with these chemical differences, COJ_W exhibited relatively greater improvement in cognitive-related behavioral performance. Although previous studies have highlighted the anti-Parkinsonian and gut-regulatory activities of OJ polysaccharides (35), these results imply that the superior effects of COJ_W may arise from the combined actions of multiple bioactive components rather than carbohydrate content alone. Instead, the observed benefits may result from the combined actions of multiple phytochemical classes, including carbohydrates, phenolic compounds, steroidal saponins, homoisoflavonoids, and other glycosylated constituents. Given that the current chemical analysis was primarily qualitative, further studies incorporating quantitative profiling and bioactivity-guided fractionation are required to identify the key bioactive components responsible for the anti-Parkinsonian effects of OJ extracts.

Chronic neuroinflammatory responses serve as a pivotal driver in the pathogenesis of various neurodegenerative disorders. MPTP promotes reactive oxygen species production, activates microglial cells, and induces the release of inflammatory mediators such as IL-6, IL-1β, and TNF-α, ultimately leading to neuronal damage. In addition to astrocytes and microglia, neurons themselves have been reported to release cytokines, thereby stimulating neuroinflammatory responses and contributing to dopaminergic neuronal dysfunction (36, 37). Neuroinflammation and α-syn exhibit a reciprocal relationship, and this interaction has been reported to accelerate the progression of PD. In this study, OJ extracts reduce α-syn protein levels in the midbrain of mice and decrease pro-inflammatory cytokines, suggesting that OJ may inhibit microglial activation, thereby prevent subsequent neuronal injury and ultimately exert a protective effect on TH^+^ neurons.

Significant gut dysbiosis is a hallmark feature of PD, with the enteric microbiome showing profound alterations relative to control individuals. The Braak's hypothesis provides a novel perspective, proposing that PD originates in the gastrointestinal tract and that non-motor symptoms precede the onset of motor manifestations. Mounting data highlights that reshaping the gut microbial landscape may offer a therapeutic avenue for ameliorate PD symptoms or associated complications. The gut microbiome exerts a profound influence on PD pathogenesis by modulating neurotransmitter production, immune signaling, and metabolite interactions (38, 39). Oral administration of edible medicinal plants firstly interacts with the digestive tract, influences the gut microbiota, which may be one of the mechanisms for their functional effects (40). Previous studies suggest that certain water-soluble components of OJ exhibit notable modulatory effects on the gut microbiota and possess significant bioactivity. A comprehensive review systematically clarified the regulatory impacts of OJ polysaccharides on the gut microbiota in various disease states (35). Importantly, OJ extract has been reported to protect intestinal epithelial barrier integrity and maintain the composition of the gut microbiota, thereby promoting systemic homeostasis (41). Our 16S rRNA sequencing revealed that MPTP induced gut dysbiosis, while OJ extracts modulated gut microbiota composition, but the patterns of regulation differed depending on extraction method and geographical origin.

At the phylum level, MPTP increased Firmicutes and Actinobacteriota, while decreasing Bacteroidota. A recent large-scale metagenomic analysis indicated similar trend in the abundance of Firmicutes and Bacteroidota in PD individuals (42). However, multiple researches suggest they may not be the most significant biomarkers in PD, as their abundance varies across different investigations (43). Research has reported increased trend in abundance of the Actinobacteriota in PD individuals (43–45), which may be related to oxidative stress or metabolic dysregulation caused by gut inflammation (46, 47). Notably, COJ_W and ZOJ_W restored Verrucomicrobiota, a phylum enriched in *Akkermansia muciniphila*, which have been reported to improve the intestinal barrier integrity and maintain gut stability, thereby contributing to improved immune function and reduced inflammation (48).

At the genus level, MPTP increased *Lactobacillus*. Family Lactobacillaceae and genus *Lactobacillus* are increased in both PD patients and animal models (49). This increase in *Lactobacillus* is often accompanied by a reduction in key anti-inflammatory taxa such as family Muribaculaceae and genus *Akkermansia*, suggesting a deviation of the microbiota structure from a healthy state. The increase in *Lactobacillus* in MPTP-treated mice should be interpreted cautiously, as *Lactobacillus* contains diverse species with strain-specific functions. Although altered *Lactobacillus* abundance has been reported in PD, its direction and biological implications remain inconsistent across studies. Therefore, the role of *Lactobacillus* in the present model requires further validation at the species or strain level, together with metabolomic analysis. Both water extracts of OJ were able to increase the abundance of *norank_f__Ruminococcaceae* and *unclassified_f__Eggerthellaceae* (Figures 5E, F). Family Ruminococcaceae has been reported to degrade complex carbohydrates (50), while family Eggerthellaceae is known to convert specific dietary polyphenols into health metabolites (51). Since water extracts of OJ are expected to contain relatively abundant hydrophilic components such as polysaccharides, these components may provide substrates for specific intestinal bacteria. However, this hypothesis requires further validation using targeted chemical profiling, *in vitro* fermentation, metagenomics, or metabolomics.

COJ_W preferentially enriched several gut microbial taxa. *Dubosiella* is considered a potential probiotic with anti-aging properties and may play a role in mitigating age-related diseases (52). Notably, the abundance of *Dubosiella newyorkensis* was markedly reduced in APP^swe^/PS^1ΔE9^ (PAP) mice exhibiting cognitive decline and aging (53). Mechanistically, *Dubosiella newyorkensis* contributes to rebalancing Treg/Th17 immune responses and alleviating mucosal barrier injury (54). *Akkermansia muciniphila* has been demonstrated to protect the intestinal mucosa, regulate immune responses and metabolic profiles, and thereby contribute positively to the functioning of the gut-brain axis (55). An animal study confirmed the beneficial of *Akkermansia muciniphila* in PD, including reduced gut permeability and neuroinflammation (56), with alleviation observed in both hippocampal and striatal inflammation. Additionally, *Akkermansia muciniphila* can alleviate cognitive deficits and cerebral amyloid-β accumulation in the APP/PS1 transgenic mouse (57). Although 16S rRNA sequencing enabled the characterization of gut microbial profiles, complementary approaches may provide additional insights into the functional consequences of the observed microbial changes. Future studies examining microbiota-derived metabolites, such as short-chain fatty acids, will help to further elucidate the metabolic relevance of these alterations.

As far as we can ascertain, this is the initial report of the pharmacological effects of OJ in PD by considering both its geographical origin and extraction method. We identified the combination of origin and extraction approach that yielded the most favorable overall outcomes. Furthermore, we conducted a preliminary exploration of the differential regulatory effects of various extracts on gut microbiota.

## 5 Conclusions

The results of this study demonstrate that OJ, as an edible medicinal plant, exerts anti-Parkinsonian effects in an MPTP-induced PD mouse model, as evidenced by improved motor performance, alleviation of anxiety-like behavior, amelioration of cognitive impairment, restoration of TH^+^ neuronal populations in the SNpc, and reduction of neuroinflammatory markers. Different geographical origins and extraction methods yielded distinct bioactive profiles, leading to differential gut microbiota modulation patterns. Notably, COJ_W exhibited superior efficacy in cognitive protection and modulation of key beneficial gut microbiota taxa (*Akkermansia, Dubosiella, Ileibacterium*, and *Christensenellaceae_R-7_group*), whereas other extracts also showed beneficial effects in specific domains such as anxiety-related behavior or microbial regulation. These findings support a gut microbiota-associated mechanism underlying the anti-Parkinsonian effects of OJ and highlight the critical role of geographical origin and extraction strategy in determining its chemical composition and biological activity. Given its safety profile and long history of dietary use, OJ, particularly COJ_W, represents a promising candidate for the development of neuro-protective functional foods or nutraceuticals targeting PD relevance to the microbiota-gut-brain axis.

## Data Availability

The data presented in this study can be found in the NCBI (https://www.ncbi.nlm.nih.gov/), under BioProject accession PRJNA1482346.
