# Supplementary material for: Differential gut microbiota modulation associated with anti-Parkinsonian effects of Ophiopogon japonicus across geographical origins and extraction methods
Source: Front Nutr. 2026 Aug 6;13:1907570. doi: 10.3389/fnut.2026.1907570 (PMC13492236; doi:10.3389/fnut.2026.1907570)
Supplement: Supplementary file 1 [file Supplementary_file_1.docx]

Supplementary Material

# 1 Supplementary Figures and Tables

## 1.1 Supplementary Tables

**Supplementary Table 1. Definitions of gait parameters.**

| Parameters | Units | Definition |
| --- | --- | --- |
| Stride | s | Time duration of one complete stride for one paw. Equal to the sum of stance duration and swing duration. |
| Stride Length | cm | The spatial length that a paw traverses through a given stride. |
| Stride Length CV | CV% | Coefficient of variation in stride length. CV was calculated from the equation: 100 × standard deviation/mean value (the variability normalized to the mean). |
| Stride Frequency | Steps per second | The cadence or the number of times each second that a paw takes a complete stride. |
| Swing Duration CV | CV% | CV was calculated from the equation: 100 × standard deviation/mean value (the variability normalized to the mean). |
| Stance Width Variability | cm | Standard deviation of stance widths in the set of recorded stance. |

**Supplementary Table 2. Chemical composition identified by UPLC/Q-TOF MS form OJ.**

| **No.** | **Rt/ min** | **Identification** | **Formula** | **MS** | **Adduct** | **Error** | **MS/MS** | **Classification** | **Ref** |
| --- | --- | --- | --- | --- | --- | --- | --- | --- | --- |
| 1 | 0.61 | Glutamic acid | C_5_H_9_NO_4_ | 146.0459 | [M−H]⁻ | 0.12 | 128.0322; 102.0552 | Amino acids | (1) |
| 2 | 0.62 | Raffinose or isomer | C_18_H_32_O_16_ | 539.1375 | [M+Cl]⁻ | 1.74 | 485.1494; 383.1173; 341.1078; 323.0931; 221.0653; 179.0539 | Oligosaccharide | (2) |
| 3 | 0.63 | Fungitetraose | C_24_H_42_O_21_ | 665.2126 | [M−H]⁻ | 2.98 | 545.1688; 485.1495; 341.1078; 179.0539 | Oligosaccharide | (1, 2) |
| 4 | 0.78 | Citric acid or isocitric acid | C_6_H_8_O_7_ | 191.0206 | [M−H]⁻ | 4.56 | 173.005; 96.9596; 87.0088 | Organic acids | (1, 2) |
| 5 | 2.45 | 3-p-Coumaroylquinic acid or isomer | C_16_H_18_O_8_ | 337.0946 | [M−H]⁻ | 5.06 | 221.0653; 191.0547; 179.0539;173.0420; 161.0446 | Phenolic acids | (2) |
| 6 | 3.36 | Caffeic acid | C_9_H_8_O_4_ | 179.0351 | [M−H]⁻ | 0.66 | 135.0414; 101.0228; 89.0326 | Phenolic acids | (1-3) |
| 7 | 4.55 | p-Coumaric acid | C_9_H_8_O_3_ | 163.0390 | [M−H]⁻ | -3.95 | 119.0335; 89.0236 | Phenolic acids | (3) |
| 8 | 5.03 | o-Coumaric acid | C_9_H_8_O_3_ | 163.0390 | [M−H]⁻ | -3.95 | 119.0489; 89.0236 | Phenolic acids | (3) |
| 9 | 5.51 | Eleutheroside E | C_34_H_46_O_18_ | 401.1807 | [M−H]⁻ | 2.79 | 579.2075; 417.1539 | Lignan glycosides | (1) |
| 10 | 7.42 | 3,4-dihydroxy-allylbenzene-4-O-β-D-glucopyranoside | C_15_H_20_O_7_ | 311.1126 | [M−H]⁻ | -3.31 | 149.0609 | Phenolic glycosides | (2, 3) |
| 11 | 8.20 | Azelaic acid | C_9_H_16_O_4_ | 187.0967 | [M−H]⁻ | -4.72 | 125.0973 | Organic acids | (3) |
| 12 | 8.30 | Ophiopogonin Q | C_56_H_92_O_28_ | 1257.5754 | [M+HCOO]⁻ | -0.25 | 1211.5833; 1079.5337; 917.4728 | Steroidal saponins | (3) |
| 13 | 8.97 | Ophiopogonin K | C_51_H_84_O_25_ | 1141.5234 | [M+HCOO]⁻ | -4.35 | 1095.5331; 1077.5219; 931.4060; 849.3619 | Steroidal saponins | (3) |
| 14 | 9.30 | Ophiofurospiside I | C_51_H_84_O_24_ | 1125.5330 | [M+HCOO]⁻ | -0.41 | 1079.5337; 1061.5060; 917.4409; 771.4087; 467.2108; 305.1549 | Steroidal saponins | (4) |
| 15 | 9.62 | Ophiopogonin H | C_56_H_92_O_29_ | 1273.5754 | [M+HCOO]⁻ | 3.74 | 1227.5424; 1095.5331; 1077.5219; 931.4532; 915.4584 | Steroidal saponins | (2) |
| 16 | 10.27 | Ophiofurospiside F | C_45_H_74_O_20_ | 979.4753 | [M+HCOO]⁻ | -0.26 | 933.4744; 915.4691; 627.3033; 609.2869; 555.2523; 409.2252 | Steroidal saponins | (3, 4) |
| 17# | 10.96 | Borneol 7-O-[β-D-apiofuranosyl-(1→6)]-β-D-glucopyranoside | C_21_H_36_O_10_ | 493.2245 | [M+HCOO]⁻ | 2.07 | 447.2245; 315.1809; 161.0446; 101.0228 | Monoterpenoid glycosides | (2, 5) |
| 18 | 11.49 | Ophiofurospiside M | C_45_H_74_O_19_ | 963.4759 | [M+HCOO]⁻ | -4.92 | 917.4835; 771.4185; 591.3533 | Steroidal saponins | (2, 5) |
| 19 | 12.24 | (22S)-cholest-5-ene-1β, 3β, 16β, 22-tetrol-1-O-α-L-rhamnopyranosyl-16-O-β-D-glucopyranoside | C_39_H_66_O_13_ | 787.4499 | [M+HCOO]⁻ | 1.72 | 741.4428; 579.3854; 433.3290 | Steroidal saponins | (2, 4) |
| 20 | 12.44 | Ophiopogonin G | C_56_H_92_O_27_ | 1241.5830 | [M+HCOO]⁻ | 1.77 | 1195.5825; 1063.5372; 1033.5205; 901.4722; 755.4164; 737.4097; 575.3585 | Steroidal saponins | (3) |
| 21 | 14.31 | (25R)-spirost-5-ene-1β,3β,23,24-tetrol-1‑O‑α‑L‑rhamnopyranosyl‑(1→2)‑[β‑D‑xylopyranosyl‑(1→3)]‑α‑L‑arabinopyranosyl-24‑O‑α‑L‑fucopyranoside | C_49_H_78_O_22_ | 1063.5028 | [M+HCOO]⁻ | 4.51 | 1017.4993; 885.4551; 739.3906; 607.3505 | Steroidal saponins | (3) |
| 22 | 15.43 | (25R)-26-O-β-D-glucopyranosyl-furost-5-ene-1β,3β,22α,26-tetraol-1-O-β-D-xylopyranosyl-(1→3)-[α-L-rhamnopyranosyl-(1→2)]-β-D-fucopyranoside | C_50_H_82_O_20_ | 1079.5222 | [M+HCOO]⁻ | -4.78 | 1033.5319; 901.4828; 755.4261; 575.3585 | Steroidal saponins | (6) |
| 23 | 19.89 | Ophiopogonin R | C_39_H_62_O_15_ | 815.4064 | [M+HCOO]⁻ | -0.83 | 769.4054; 623.3414; 443.2796 | Steroidal saponins | (6) |
| 24 | 20.34 | (25R)-spirost-5-ene-3β,14α,17α-triol-3-O-α-L-rhamnopyranosyl-(1→4)-[β-D-xylopyranosyl-(1→3)-α-L-arabinopyranosyl-(1→2)]-β-D-glucopyranoside | C_49_H_78_O_22_ | 1063.5028 | [M+HCOO]⁻ | 4.51 | 1017.4993; 885.4551; 753.4060; 289.0923; 89.0236 | Steroidal saponins | (3, 4) |
| 25# | 20.81 | Cixiophiopogon A or Ophiopojagonin C | C_44_H_70_O_18_ | 931.4532 | [M+HCOO]⁻ | -1.31 | 885.4551; 753.4050; 607.3505; 445.2982; 205.0699; 89.0236 | Steroidal saponins | (4, 6) |
| 26# | 21.10 | ophiopogonin Ra | C_39_H_62_O_14_ | 799.4099 | [M+HCOO]⁻ | -2.83 | 753.4050; 607.3505; 445.2982; 205.0699 | Steroidal saponins | (4-6) |
| 27# | 21.76 | Ophiopogonside A | C_26_H_44_O_10_ | 561.2916 | [M+HCOO]⁻ | -0.09 | 515.2889; 101.0228 | Steroidal saponins | (4) |
| 28 | 22.55 | (25R)-Ruscogenin-1-O-β-D-glucopyranosyl-(1→2)-β-D-xylopyranosyl-(1-3)-β-D-fucopyranoside | C_44_H_70_O_17_ | 869.4571 | [M+HCOO]⁻ | 3.53 | 737.4103; 719.4041; 591.3533 | Steroidal saponins | (2) |
| 29# | 23.44 | Deacetyl ophiopojaponin A | C_44_H_70_O_17_ | 915.4584 | [M+HCOO]⁻ | -1.21 | 869.4571; 753.4051; 737.4198; 205.0699 | Steroidal saponins | - |
| 30# | 23.68 | 14‑Hydroxy sprengerinin C | C_44_H_70_O_17_ | 915.4584 | [M+HCOO]⁻ | -1.21 | 869.4571; 737.4198; 205.0699 | Steroidal saponins | - |
| 31 | 24.19 | 14-hydroxy diosgenin 3-O-α-L-rhamnopyranosyl-(1→2)-β-D-glucopyranoside | C_39_H_62_O_13_ | 783.4142 | [M+HCOO]⁻ | -3.89 | 737.4103; 591.3533 | Steroidal saponins | (6) |
| 32 | 28.91 | Ruscogenin 3‑O‑acetyl‑α‑L‑rhamnopyranosyl-(1→4)-α‑L‑rhamnopyranosyl-(1→2)-β‑D‑glucopyranoside | C_41_H_64_O_13_ | 825.4302 | [M+HCOO]⁻ | 2.89 | 799.4283; 737.4103; | Steroidal saponins | (2) |
| 33 | 29.31 | Ophiopogonin P" | C_41_H_64_O_13_ | 825.4302 | [M+HCOO]⁻ | 2.89 | 799.4283; 737.4103; | Steroidal saponins | (2) |
| 34 | 31.15 | Pennogenin-3-yl 2-O-acetyl-α-L-rhamnopyranosyl-(1→2)-β-D-glucopyranoside | C_41_H_64_O_13_ | 825.4302 | [M+HCOO]⁻ | 2.89 | 799.4283; 737.4103; 591.533 | Steroidal saponins | (6) |
| 35# | 31.59 | Methylophiopogonone A | C_19_H_16_O_6_ | 339.0885 | [M−H]⁻ | 3.20 | 311.094; 217.0477; 189.0549; 131.0496 | Homoisoflavonoids | - |
| 36# | 31.82 | Ophiopogonin D | C_44_H_70_O_16_ | 899.4648 | [M+HCOO]⁻ | 0.23 | 853.4608; 721.4169; 575.3585 | Steroidal saponins | - |
| 37 | 32.32 | Sprengerinin C | C_44_H_70_O_16_ | 899.4648 | [M+HCOO]⁻ | 0.23 | 853.4608; 721.4169; 575.3585 | Steroidal saponins | (2, 3, 6) |
| 38# | 32.80 | Methylophiopogonone B | C_19_H_18_O_5_ | 325.1087 | [M−H]⁻ | 1.70 | 309.0728; 281.0792; 203.0331; 145.0265; 117.033 | Homoisoflavonoids | - |
| 39 | 32.81 | 5-hydroxy-7,4′-dimethoxy-6,8-dimethyl homoisoflavanone | C_20_H_22_O_5_ | 341.1403 | [M−H]⁻ | 2.50 | 220.0718; 205.0699; 193.0502 | Steroidal saponins | (3) |
| 40# | 33.31 | Ophiopogonin D' | C_44_H_70_O_16_ | 899.4648 | [M+HCOO]⁻ | 0.23 | 853.4608; 721.4169; 575.3585 | Steroidal saponins | - |
| 41# | 33.54 | Methylophiopogonanone A | C_19_H_18_O_6_ | 341.1013 | [M−H]⁻ | -5.05 | 291.1973; 205.0497; 178.0613; 149.0222; 135.0414 | Homoisoflavonoids | (2, 3) |
| 42 | 33.75 | Ophiopogonin B | C_39_H_62_O_12_ | 767.4242 | [M+HCOO]⁻ | 2.43 | 721.4169; 575.3585; 439.3002 | Steroidal saponins | (6) |
| 43# | 34.20 | Liriopesides B | C_39_H_62_O_12_ | 767.4242 | [M+HCOO]⁻ | 2.43 | 721.4169; 575.3585; 439.3002; 311.1685; 119.0335 | Steroidal saponins | - |
| 44# | 35.04 | Methylophiopogonanone B | C_19_H_20_O_5_ | 327.1242 | [M−H]⁻ | 1.23 | 206.0542; 178.0613; 149.0222; 121.0277 | Homoisoflavonoids | - |
| 45 | 36.14 | Ophiopogonin C′ | C_39_H_62_O_12_ | 767.4242 | [M+HCOO]⁻ | 2.43 | 721.4169; 575.3585; 439.3002 | Steroidal saponins | (3) |
| 46 | 36.48 | Ophiopogonin P' | C_46_H_72_O_17_ | 941.4731 | [M+HCOO]⁻ | -2.19 | 895.4780; 853.4608; 835.4543; 721.4169 | Steroidal saponins | (2, 3) |
| 47 | 38.80 | (25R)-spirost-5-ene-1β,3β-diol-3-O-acetyl-(1→3)-α-L-rhamnopyranosyl-(1→2)-α-L-fucopyranoside | C_41_H_64_O_13_ | 809.4355 | [M+HCOO]⁻ | 3.21 | 763.4208; 721.4169; 703.4121; 575.3585 | Steroidal saponins | (2-4) |
| 48 | 38.82 | Ophiopogonin Q' | C_46_H_72_O_17_ | 941.4731 | [M+HCOO]⁻ | -2.19 | 895.4780; 853.4608; 835.4543; 721.4169 | Steroidal saponins | (2, 3) |
| 49 | 39.76 | Ophiopogonin A | C_41_H_64_O_13_ | 809.4355 | [M+HCOO]⁻ | 3.21 | 763.4208; 721.4169; 703.4028 | Steroidal saponins | (2) |
| 50# | 39.82 | 6-Aldehydo-isoophiopogonone A | C_19_H_14_O_7_ | 353.0648 | [M−H]⁻ | -4.88 | 325.0706; 297.0706; 203.0331; 183.0094; 119.0489 | Homoisoflavonoids | - |
| 51# | 40.40 | 8-Formyl ophiopogonone B | C_19_H_16_O_6_ | 339.0885 | [M−H]⁻ | 3.20 | 183.0094; 79.9558 | Homoisoflavonoids | - |
| 52# | 40.47 | Ophiopogonanone C | C_19_H_16_O_7_ | 355.0854 | [M−H]⁻ | -8.65 | 193.0111; 79.0173 | Homoisoflavonoids | - |
| 53 | 40.52 | Ophiopogonone C | C_19_H_14_O_7_ | 353.0648 | [M−H]⁻ | -4.88 | 325.1849; 291.1973; 277.1852; 174.9554; 183.0094 | Homoisoflavonoids | (3) |
| 54# | 40.52 | 6-Aldehydo-isoophiopogonanone A | C_19_H_16_O_7_ | 355.0854 | [M−H]⁻ | -8.65 | 353.0648; 325.0706; 183.0094; 79.0173 | Homoisoflavonoids | - |
| 55 | 40.81 | (25S)-ruscogenin1-O-2,3-O-diacetyl-α-L-rhamnopyranosyl-(1→2)-[β-D-xylopyranosyl-(1→3)]-β-D-fucopyranoside | C_48_H_74_O_18_ | 983.4902 | [M+HCOO]⁻ | 4.55 | 937.4910; 895.4780; 853.4608; 835.4543; 721.4169; 707.4050 | Steroidal saponins | (2) |
| 56# | 41.17 | 8-Formyl-ophiopogonanone B | C_19_H_18_O_6_ | 341.1013 | [M−H]⁻ | -5.17 | 193.0111; 164.075 | Homoisoflavonoids | - |

\# identified with reference substances

## 1.2 Supplementary Figures

**Supplementary Figure 1.** Safety evaluation of OJ extracts (n = 6). (A) Body weight of mice during OJ administration. (B) H&E staining of heart, liver, spleen, lung, and kidney of mice in different groups.

**Supplementary Figure 2.** Biochemical analysis of serum samples of mice treated with OJ extracts (n=6). (A) WBC: white blood cells; (B) RBC: red blood cells; (C) HGB: hemoglobin; (D) MCV: mean corpuscular volume; (E) MCH: mean corpuscular hemoglobin; (F) MCHC: mean corpuscular hemoglobin concentration; (G) PLT: platelets; (H) HCT: hematocrit; (I) AST: aspartate aminotransferase; (J) ALT: alanine aminotransferase; (K) TP: total protein; (L) GLB: globulin; (M) TBIL: total bilirubin test; (N) BUN: blood urea nitrogen; (P) ALB: albumin; (P) CREA: creatinine; (Q) direct Bilirubin; (R) IBIL: indirect Bilirubin.

**References**

1.	Zha XY, Li GW, Zhang L, Chen Q, Xia Q. Identification of Active Compounds in Ophiopogonis Radix from Different Geographical Origins by UPLC-Q/TOF-MS Combined with GC-MS Approaches. *Open Life Sci* (2022) 17(1):865–80. doi: 10.1515/biol-2022-0096.

2.	Lv YH, Xu XK, Wei YP, Shen YH, Chen W, Wei XT, et al. Characterization and Discrimination of Ophiopogonis Radix with Different Levels of Sulfur Fumigation Based on UPLC-QTOF-MS Combined Molecular Networking with Multivariate Statistical Analysis. *Metabolites* (2023) 13(2). doi: 10.3390/metabo13020204.

3.	Lyu CG, Kang CZ, Kang LP, Yang J, Wang S, He YL, et al. Structural Characterization and Discrimination of Ophiopogon Japonicas (Liliaceae) from Different Geographical Origins Based on Metabolite Profiling Analysis. *J Pharm Biomed Anal* (2020) 185:113212. doi: 10.1016/j.jpba.2020.113212.

4.	Jin X, Zhang JQ, Li Y, Wu WY, Zhang H, Yao CL, et al. Nontargeted Metabolomic Analysis and Multiple Criteria Decision-Making Method Induced Robust Quality Markers Screening for the Authentication of Herbal Medicines from Different Origins by Taking Ophiopogon Japonicus (L. F.) Ker-Gawl. As a Case Study. *J Sep Sci* (2021) 44(7):1440–51. doi: 10.1002/jssc.202000655.

5.	Wang Y, Shao HF, Lyu C, Park KH, Nguyen TK, Yang IJ, et al. Ophiopogon Japonicus Root Extract Attenuates Obesity-Induced Muscle Atrophy through Regulation of the PI3K-AKT-mTOR/FoxO3a Signaling Pathway and Lipid Metabolism in Mice and C2C12 Myotubes. *Nutrients* (2025) 17(24). doi: 10.3390/nu17243946.

6.	Ling Y, Tang YH, Xu YY, Zhang Q, Zhang C, Zhang YY, et al. Rapid Screening and Identification of Chemical Constituents from Ophiopogon Japonicus by High-Performance Liquid Chromatography Coupled to Electrospray Ionization and Quadrupole Time-of-Flight Mass Spectrometry. *J Chromatogr Sci* (2020) 58(7):641–50. doi: 10.1093/chromsci/bmaa029.
